# Breast Cancer in Pregnant Young Women: Clinicopathological Profile, Survival, and Pregnancy Outcomes

**DOI:** 10.7759/cureus.47578

**Published:** 2023-10-24

**Authors:** Nicolas Ramírez-Torres, Rodolfo Rivas-Ruiz, Alfonso Reyes-López

**Affiliations:** 1 Oncological Gynecology, High Specialty Medical Unit from Gynecology and Obstetrics Hospital No. 3, National Medical Center (NMC) La Raza, Mexico City, MEX; 2 Pediatrics, Clinical Research Division, Clinical Research Training Center, Mexico City, MEX; 3 General Practice, Center for Economic and Social Studies in Health, Hospital Infantil “Federico Gómez”, Mexico City, MEX

**Keywords:** cancer, retrospective cohort, survival, breast cancer, pregnancy

## Abstract

Background

Breast cancer is one of the most common cancer types diagnosed during pregnancy; the presence of any neoplasm in pregnant women faces clinical dilemmas and challenges in cancer and pregnancy management. Pregnancy-associated breast cancer (PABC) is defined as breast cancer diagnosed during pregnancy or within one year after delivery. The aim of this study was to describe tumor clinicopathological characteristics and pregnancy outcomes in PABC patients.

Materials and methods

This is a retrospective cohort assessing PABC patients. Qualitative variables were compared using Fisher’s exact test. Kaplan-Meier method was used to calculate survival. Cox regression and logistic regression methods were used to estimate the hazard ratio (HR) and odds ratio (OR), respectively.

Results

We assessed 16 PABC patients. Women ≤ 35 years of age were mainly diagnosed at advanced stage (88.8%) with ER-negative disease (77.8%). Patients with >4 pathological lymph nodes (25%; p = 0.001) and ER-negative disease (50%; p = 0.646) showed poor five-year overall survival (OS). In the multivariate analysis, nodal involvement was the main predictor associated with poorer OS (HR = 1.4, 90% confidence interval [CI]: 1.14 to 1.8).

The following risk factors might influence the risk of preterm delivery: maternal older age, gestational age at diagnosis, and intrauterine exposure to chemotherapy, but an adjusted OR of 0.61 (90% CI: 0.34 to 1), 0.80 (90% CI: 0.66 to 0.9), and 0.013 (90% CI: 0.00 to 0.9), respectively, did not statistically support such an effect.

Conclusions

Younger women with PABC had a more aggressive pathological profile that might partly explain the poor OS. Obstetrical adverse events related to preterm delivery should be avoided with better planning of specialized strategies.

## Introduction

Breast cancer is one of the most common cancer types diagnosed among pregnant women. Obstetricians are seeing a higher number of pregnancy-associated breast cancer (PABC) cases [1-3] due to the upward trend of childbearing delay after the third decade of life combined with the increased incidence of breast cancer worldwide [2-4].

PABC is defined as breast cancer diagnosed during pregnancy and/or within one year after delivery [5-9]. This entity is uncommon, which is estimated to range from 1/3,000 to 1/10,000 pregnancies, in developed countries; the incidence increases and ranges from 15 to 44 per 100,000 pregnancies [2-5]. We face clinical dilemmas and challenges in cancer management [5,6] with the presence of any neoplasm in pregnant women, which is usually associated with large tumors and nodal involvement [9-16].

Gestational physiologic changes increase mammary nodularity and engorgement, which are progressive until childbirth. These transient and permanent structural changes of breast tissue might mask signs and symptoms of early breast cancer, making the exploration of early breast cancer difficult [2,5,7,11-13,16] and limiting the sensitivity of the mammography and ultrasound [5,6,13].

Although most mammary tumors are benign in young women, we should avoid the misdiagnosis of any breast mass as it might contribute to a delayed diagnosis of malignant mammary tumors [5,6]. On the other hand, the prognosis remains a subject of debate due to conflicting outcomes in PABC studies [2-4,6-16]. Many studies have noted that poor prognosis is the result of aggressive tumor characteristics themselves [4-6,10-16]. The prognosis may also change according to the hospital centers' resources, with better prognosis in high-income and worse prognosis in low-income hospitals.

There are only a few published cohorts of Latin-American patients with PABC, so the aim of this study was to describe tumor characteristics in PABC patients, survival, and neonatal outcomes. The information might help any physician to begin a prompt intervention. PABC patients should be referred to specialist physicians for breast and pregnancy.

This article was published as a preprint at https://www.medrxiv.org/content/10.1101/2022.09.29.22280276v1.full-text (Original article: Nicolás R-T, Rodolfo R-R, Alfonso R-L, Kingston U-W. Breast Cancer in Pregnant Young Women: Clinicohistological Profile, Risk of Death and Pregnancy Outcomes; September 30, 2022).

## Materials and methods

Study population

This research evaluates a retrospective cohort of PABC patients. Young women aged ≤40 years with unilateral breast cancer were included. We summarized the experience in our hospital between March 1992 and June 2010. All cases were under the care of a multidisciplinary physician team (composed of oncologists, obstetricians, anesthesiologists, internists, and neonatologists) who serve at the Medical Unit of High Specialty of Gynecology and Obstetrics Hospital No. 3, “La Raza” National Medical Center, belonging to the Mexican Institute of Social Security. It is a tertiary referral center for the care of pregnant women and breast cancer management, which explains the higher frequency of cases.

Clinicopathological factors and oncological management

Epidemiological and clinicopathological data, as well as immunohistochemistry (IHC) retrospective information for the estrogen receptor (ER), progesterone receptor (PgR), and human epidermal growth factor receptor 2 (HER2) status, were extracted from medical records, which have been previously described in detail [17]. Imaging studies for diagnosis and staging were carried out according to standard guidelines in order to ensure fetal health. Clinical staging (I to III) was based on the American Joint Committee on Cancer (AJCC)'s tumor, node, and metastasis (TNM) classification (sixth edition) [18]. TNM stage established prior to 2002 was restaged.

Nottingham prognostic index (NPI) was established when tumor size, number of lymph nodes, and grade were combined [12]. Standardized oncological management (chemotherapy, definitive surgery, and radiotherapy), course of pregnancy, and perinatal outcomes have been previously described [17]. At that time, patients with positive ER- or PgR disease did not receive endocrine therapy nor did patients with HER2+ tumors receive trastuzumab, and breast cancer subtypes were not considered. Subsequently, the molecular classification by IHC of breast cancer was established. Later, our hospital carried out a retrospective IHC study only for ER, PgR, and HER2. Molecular classification by IHC was described according to commonly accepted definitions [19,20]. Five subtypes have been described: luminal A-like (LA: ER+, PgR+, HER2), luminal B-like HER2-negative (LB/HER2-: ER+, PgR+/-, HER2-), luminal B-like HER2-positive (LB/HER2+: ER+, PgR+/-, HER2+), HER2+ (ER-, PgR-, HER2+), and triple-negative (TN) (ER-, PgR-, HER2-).

Statistical analysis

The primary objective was to describe tumor clinicopathological characteristics and newborn outcomes. The secondary objective was to assess the disease-free survival (DFS) and overall survival (OS) in PABC patients.

Qualitative variables were compared using Fisher’s exact test. The median and interquartile range (IQR: 25th-75th) were evaluated using nonparametric tests for age, tumor size, NPI, and gestational age. The Kaplan-Meier method was used to construct survival curves and compare them with the log-rank test. Alive patients without events were censored at the time of the last follow-up evaluation.

Multivariate models were performed to estimate the hazard ratio (HR), confidence intervals (CI), and regression coefficient (b) using Cox proportional hazards regression [21]. We hypothesized that more aggressive clinicopathological factors are more common in younger PABC patients. Interactions between variables were not explored.

Delivery (term or preterm) was associated with pregnancy-related risk factors using multivariate logistic regression; the odds ratio (OR) was estimated with the null hypothesis of no relationship. All tests were two-sided; p-value < 0.05 was considered statistically significant. A significance level of 10% was used in both Cox regression and logistic regression. STATA version 14 (Stata Corp., College Station, TX) was used for all statistics. The study was assessed and approved by the Institutional Review Board of National Medical Center, La Raza.

## Results

Patient characteristics by age group

Sixteen PABC patients were evaluated. Baseline characteristics are shown in Table 1. Most pregnant women aged ≤35 years were diagnosed at clinical stage IIIA-B (88.8%), with axillary lymph node involvement (100%) and NPI score > 5.4 (66.7%). As for hormonal status, tumors classified as ER- and PgR-negative were more common (77.8% and 100%, respectively), and only one-third were HER2+ tumors, although the differences did not reach statistical significance (all, p > 0.05), except for PgR (p = 0.019), as shown in Table 2.

**Table 1:** Baseline characteristics of pregnant women IQR: Interquartile range.

Characteristics	Group	n	(%)
Age of patient	<35 years	9	56.2
	>35 years	7	43.7
Familiar cancer history	First grade	3	18.7
First pregnancy (>29 years)	>29 years	7	43.7
Parity at diagnosis	0	3	18.7
	1	5	31.2
	2-3	7	43.7
	>4	1	6.3
Trimester at diagnosis	First	7	43.7
	Second	5	31.2
	Third	4	25.0
Upfront chemotherapy (trimester)	Second/third	9	56.2
	After delivery	7	43.7
Birth weight of newborn	<2500 g	9	56.2
	>2500 g	4	25.0
	Miscarriage	3	18.7
Maternal characteristics associated with breast cancer		Median	IQR (25^th^, 75^th^)
Maternal age at diagnosis (years)		35	34, 36
Evolution time of tumor at diagnosis (months)		6	3, 9
Gestational age at diagnosis (weeks)		17	8, 31
Gestational age at delivery (weeks)		37	32, 38
Gestational age at upfront chemotherapy (weeks)		21	20, 30

**Table 2:** Clinical-pathological characteristics by age group IDC: Invasive ductal carcinoma; MC: Mixed carcinoma; ER: Estrogen receptor; PgR: Progesterone receptor; HER2: Human epidermal growth factor receptor 2; NPI: Nottingham prognostic index; Ref: Reference; P*: Fisher’s exact test.

		Age, <35 years	Age, >35 years	P*
Characteristics	Status	n = 9 (%)	n = 7 (%)	
Tumor size (T)	T2-3	8 (88.8)	4 (57.1)	0.145
	T4	1 (11.1)	3 (42.8)	
Axillary node (N)	N0	0 (0.0)	1 (14.3)	0.240
	N1-2	9 (100)	6 (85.7)	
Clinical stage	IIA-B	1 (11.1)	2 (28.5)	0.550
	IIIA-B	8 (88.8)	5 (71.4)	
Tumor grade (G)	G2	4 (44.4)	5 (71.4)	0.280
	G3	5 (55.8)	2 (28.5)	
Histology	IDC	7 (77.2)	5 (71.4)	Ref.
	MC	2 (22.2)	1 (14.3)	0.792
	Medullar	0 (0.0)	1 (14.3)	0.260
ER	Positive	2 (22.2)	5 (71.4)	0.072
	Negative	7 (77.7)	2 (28.5)	
PgR	Positive	0 (0.0)	4 (57.1)	0.019
	Negative	9 (100)	3 (42.8)	
HER2	Negative	6 (66.6)	5 (71.4)	1.000
	Positive	3 (33.3)	2 (28.5)	
NPI (score)	<5.4	3 (33.3)	4 (57.1)	0.615
	>5.4	6 (66.6)	3 (42.8)	

Survival according to prognostic factor

**Figure 1:**
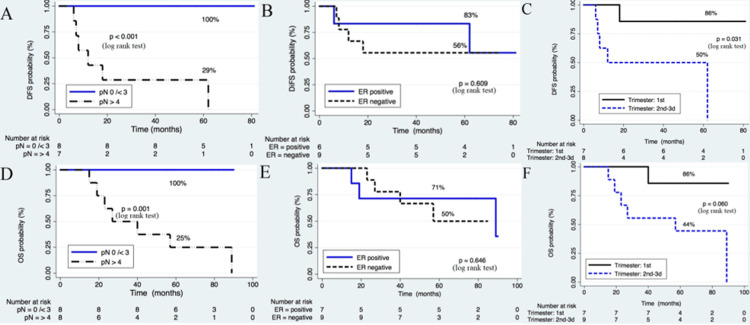
Kaplan-Meier curves according to pathological lymph nodes (pNs), estrogen receptor (ER) status, and trimester of pregnancy at diagnosis of breast cancer. (A-C) Disease-free survival (DFS) and (D-F) overall survival (OS).

For all patients, the median follow-up was 47.5 months (range: 0-81) for DFS and 64.5 months (range: 15-90) for OS. The survival curves are shown in Figure 1 (Panels A-F). As for pathological lymph nodes (pNs), five-year DFS was 100% for patients with 0-3 pNs and 29% for those with >4 pNs (p < 0.001), whereas five-year OS was 100% and 25% (p = 0.001) for the same pNs groups, respectively, as shown in Figure 1 (Panels A and D). Thus, the higher number of pNs influenced negatively on survival.

Patients with ER-negative tumors showed a trend toward poorer five-year survival than for those with ER-positive tumors, DFS was 56% vs. 83% (p = 0.609) and OS was 50% vs. 71% (p = 0.646), respectively. However, DFS and OS were better in patients with RE-positive disease. The differences did not reach statistical significance (Figure 1, Panels B and E).

Patients in whom cancer was diagnosed during the first (1st) trimester of pregnancy had a significantly better DFS in comparison with those diagnosed during the second (2nd) and third (3rd) trimesters (five-year DFS: 86% vs 50%, respectively; p = 0.031). Although there was an improved OS for the first-trimester group (five-year OS: 86% vs. 44%, respectively; p = 0.060), the difference found was not statistically significant (Figure 1, Panels C and F).

Mortality risk

Bivariate analyses showed that only lymph node involvement and NPI had an independent predictive effect on OS; they experienced an increase in the risk of death of 35% (HR = 1.35, 90% CI: 1.13 to 1.6) and 90% (HR = 1.9, 90% CI: 1.10 to 3.2), respectively (Table 3).

**Table 3:** Cox regression analysis of covariables affecting OS in pregnant women with breast cancer OS: Overall survival; b: Regression coefficient; SE: Standard error; HR: Hazard ratio; CI: Confidence interval; pN: Pathological lymph node; ER: Estrogen receptor; NPI: Nottingham prognostic index; trimester of pregnancy at diagnosis^£^: first vs. second and third; HR^£^: Unadjusted; HR*: Adjusted for pN, age, tumor, and ER.

	Bivariate analysis				Multivariate analysis			
Covariable	b	SE	HR^£ ^(90% CI)	p	b	SE	HR* (90% CI)	p
pN (number)	0.301	0.107	1.35 (1.13-1.6)	0.005	0.367	0.144	1.44 (1.14-1.8)	0.011
Age (years)	-0.245	0.131	0.78 (0.63-0.9)	0.062	-0.224	0.137	0.79 (0.63-1.0)	0.102
Tumor (cm)	0.006	0.160	1.00 (0.77-1.3)	0.969	-0.253	0.346	0.77 (0.43-1.3)	0.463
ER-negative	0.398	0.872	1.48 (0.35-6.2)	0.648	-0.598	1.309	0.54 (0.06-4.7)	0.648
NPI (score)	0.638	0.329	1.90 (1.10-3.2)	0.052				
Trimester^£^	1.788	1.082	5.98 (1.01-35)	0.099				

In the multivariate analyses, after adjusting for selected prognostic factors, only lymph node involvement was significantly associated with OS: For each additional positive lymph node in the number of pNs, there was a 44% increased risk of death (HR = 1.44, 90% CI: 1.14 to 1.8) in comparison to lower number of pNs. The influence of the other variables on OS was less clear (Table 3).

Risk of preterm birth

**Figure 2:**
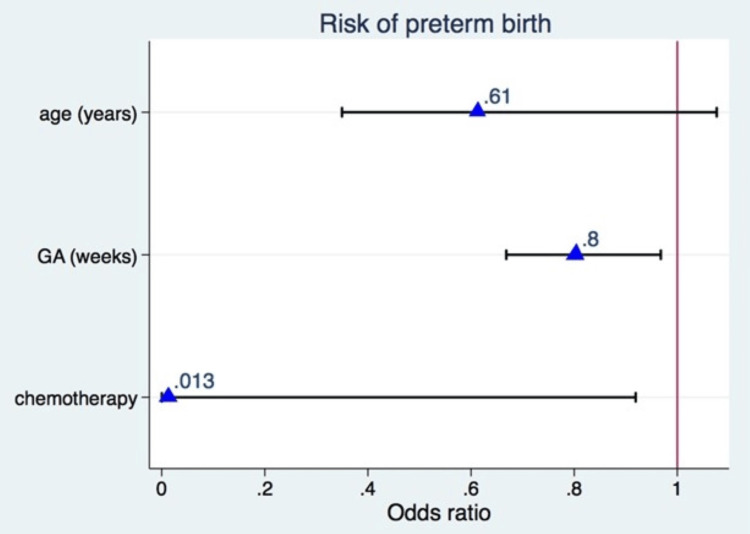
Forest plot of logistic regression for preterm birth adjusted for mother's age, gestational age (GA) at diagnosis, and chemotherapy exposure (postpartum vs intrauterine) in women with breast cancer

The following risk factors could influence an increase in the risk of preterm birth: maternal older age, gestational age at diagnosis, and intrauterine chemotherapy exposure, but their adjusted ORs of 0.61 (90% CI: 0.34 to 1.1), 0.80 (90% CI: 0.66 to 0.9), and 0.013 (90% CI: 0.00 to 0.9), respectively, did not statistically support such effect, maybe due to a statistically underpowered sample (Figure 2). Most cases (77%) exposed to chemotherapy during pregnancy had a full-term pregnancy with a live birth.

Follow-up

Follow-up compromised a rate of 939 months per person; during this time frame, there were seven deaths in PABC patients with locally advanced stages (IIB to IIIB). In this group, there were four grade 3 tumors. The systemic disease mainly occurred in HER2+ (four out of five) and TN tumors (three out of six).

Among nine PABC patients exposed to chemotherapy during pregnancy, one-third had systemic recurrences after chemotherapy completion, and only one case experienced progression and death during the treatment with pregnancy termination. All patients (seven) with systemic disease have died.

## Discussion

Extensive research has been addressed in improving the knowledge about tumor characteristics and oncological management [7-17], which has allowed the establishment of better obstetrical-fetal care favoring the health of the mother-child dyad [5,6,8,22].

In this study, one-third (37.5%) of women aged ≤30 years had delayed their first pregnancy; in this group, half were nulliparous women. No pregnant patient reported an induced abortion or a pregnancy termination. A family history of breast cancer was not associated with PABC as only three cases had this characteristic [17].

Prognostic factors are often taken into account for establishing the prognosis [23]. The TNM stage is one of the most important parameters that influence survival; breast cancer during pregnancy is often diagnosed at a locally advanced stage (IIb or more advanced), accounting for 39%-90% of cases [6,7,10-15,17], which is associated with a higher risk of recurrence and death.

In this cohort, all women self-detected a palpable breast mass, with nearly all tumors being diagnosed at a locally advanced stage (93.7% at stages IIB to IIIB). Patients with larger tumors had a higher mortality risk, but unadjusted HR reduced its magnitude from 1.0 to 0.77 (90% CI: 0.43 to 1.3) after multivariate adjustment. Thus, tumor size did not influence the risk of death, perhaps due to a lack of variability, as three-quarters of the patients had tumors larger than 5 cm. It is obvious that the prognosis becomes poor when increasing the tumor size, particularly stage III entails a poorer prognosis.

Lymph node involvement often occurs in PABC patients in more than two-thirds of cases (50%-93.7%) [4,7-17]; this factor is considered the strongest predictor in the prognosis. In our cohort, ipsilateral axillary region lymph node involvement was a relatively common clinical finding (93.7%). The number of pNs was stratified after neoadjuvant chemotherapy (NC) followed by surgery or surgery alone; half of the cases had <3 pNs, and the other half (>4 pNs) showed a high percentage of systemic recurrences (87.5%, seven out of eight). Unadjusted HR for nodal involvement slightly increased from 1.35 to 1.44 after the multivariate adjustment. Therefore, patients with a higher number of pNs were associated with an increased mortality rate.

Young age at diagnosis has a strong negative effect on survival [12-15,17,23-25], and most PABC patients are young women [7-17]. PABC patients compared with non-PABC women are more likely to be diagnosed at a younger age, with advanced T/N stages, high grade, and ER- and PgR-negative tumors [5-7,15-17]. TN or HER2+ tumors [4,8,9,12,25,26] and BRCA1/2 gene mutations [25] are far more common in younger women. Therefore, the high expression of aggressive biopathological characteristics of the tumor is established by the young age rather than the pregnancy itself.

In this study, we found an increased mortality rate (56%) in women aged ≤35 years in comparison to those aged >35 years (29%); perhaps, this is influenced by the delayed tumor diagnosis. However, the HR did not change substantially, neither before nor after adjustment (HR = 0.79, 90% CI: 0.63 to 1.0). Thus, age did not act as the main driver in the risk of death, perhaps due to a relatively small number of patients at maternal older age involved in this cohort.

Other tumor features have been described for PABC women, and the predominant histology is infiltrating ductal carcinoma (accounting for 75%-93%) [2-4,6-15,17], whereas medullary breast cancer is infrequent (2.7%-6.2%) [4,13]. Most studies have reported higher-grade tumors, with an incidence ranging from 48% to 84% [4,7-9,11,12,15,17], and lymphovascular invasion is identified in 21%-67.3% of cases [7-9,11,12,17].

Most studies have found a high proportion of ER- (45%-62%) and PgR-negative tumors (35%-59%) in PABC patients [4,6-9,13-17,26], which are commonly associated with a poor prognosis [4,10,12,15,16,26].

In this study, although ER-negative tumors were far more common (77.8%) in the subgroup of women younger than 35 years of age, the mortality rate was similar in patients with ER-negative and ER-positive tumors (44% vs 43%, respectively). Unadjusted HR for ER-negative reduced its magnitude from 1.48 to 0.54 (90% CI: 0.06 to 4.7) after multivariate adjustment. Thus, we found no association between the ER status and mortality rate.

HER2 overexpression (17%-65%) [4,7-9,11-17,26] and high levels of ki-67 (39%-100%) [8,9,12-15] are reported to have wide variability. Few studies have reported the p53 abnormal expression, which accounts for 48%-68.3% [12,13]; in view of this inconsistent data, we should continue evaluating these IHC biomarkers in PABC.

The main IHC biomarkers (ER, PgR, HER2, and ki67) have been incorporated to investigate the molecular subtype [8,9,11,13-15,26]. Young women with PABC often have TN (28%-50%) or HER2+ tumors [2,7-9,12,15,16]. Some Asian [13,14] and European [11,12] studies have reported that luminal B tumors are one of the most common breast cancer subtypes diagnosed during pregnancy. Thus, the molecular subtype is different by age, ethnic background, and geographical region [25].

**Figure 3:**
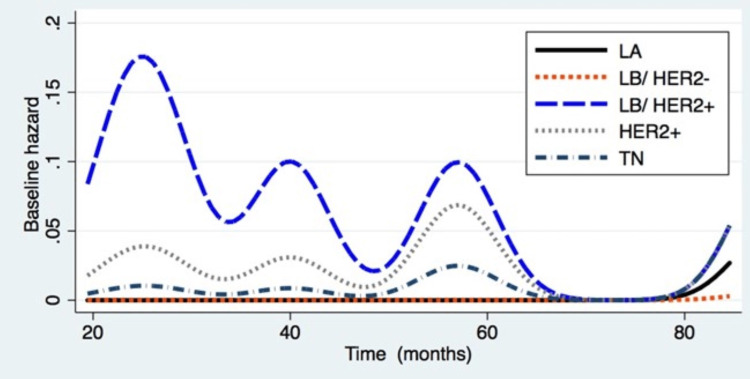
Mortality according to molecular subtype LA: Luminal A; LB: Luminal B; HER2: Human epidermal receptor factor 2; TN: Triple negative.

In this study, the commonly found molecular subtypes were TN (37.5%) and luminal A (25%) tumors, but patients with HER2+ tumors (of the LB subtype or not) showed a higher mortality rate, with three critical points in terms of the mortality rate being observed, one with the highest rate at 25 months and two with the lowest rates at 40 and 55 months for both HER2+ subtypes (Figure 3).

A considerable number of case-control studies [8-12,14,15], population-based cohorts [2-4,16,24], and meta-analysis studies [27,28] have determined a higher mortality risk if breast cancer is diagnosed during pregnancy. Wide research suggests that tumors diagnosed at all time frames of the postpartum period have exhibited greater differences in survival, even though the risk of death is more pronounced for those diagnosed within the first 12 months of the postpartum period in comparison with non-pregnancy-related breast cancer cases [2,4,24,27-30]. Some consider that the postpartum period can extend up to five or 10 years after the delivery [4,28-30]. Pregnancy and postpartum physiological events are intertwined, but they should be separated because there are vast clinical and biological differences [4,24,27-30].

In case-control studies, Rodriguez et al. [10] and Liao et al. [14] noted that PABC patients when compared to controls had a higher risk of death of 14% (HR = 1.14, 95% CI: 1.0 to 1.29) and 23% (HR = 1.23, 95% CI: 0.46 to 3.29), respectively. Azim et al. [11] also reported a higher risk of death in pregnant women (HR = 2.6, 95% CI: 1.0 to 6.5).

In a meta-analysis that included 45 studies, Amant et al. [28] evaluated DFS and OS in 6,602 cases and 157,657 controls. PABC patients in comparison with controls showed a significantly higher risk of death, with a pooled hazard ratio (pHR) of 1.45 (95% CI: 1.30 to 1.63); the heterogeneity was significant (I^2^ = 64.9; p < 0.001). Mortality was significant for those diagnosed 12 months after their last delivery (pHR = 1.59, 95% CI: 1.3 to 1.82). Conversely, mortality was not significantly different 70 months after the last delivery (pHR = 1.14, 95% CI: 0.99 to 1.25).

In contrast, other studies found similar survival rates in PABC patients compared to the nonpregnant control group [6-9,24] after matching by age and stage. For example, O’Sullivan et al. [6] showed a five-year OS of 75% and 81%, and in the trial by Ploquin et al. [8], the five-year OS was 83.1% and 85.5% (all, p > 0.05). Instead, Litton et al. [7] found opposing results: OS was slightly superior in PABC women in comparison with controls (77% vs 71%, respectively; p = 0.046). It is not exactly clear why pregnant patients had better outcomes.

Young age, lymph node involvement, and advanced stage are not the only factors implicated in generating a poor prognosis. Mechanisms causing PABC are unclear due to hormonal and immune alterations induced during pregnancy or the microenvironment of the postpartum breast involution as they might be implicated by exerting an adverse effect on the stroma of the breast tissue, favoring the proliferation of mammary stem cells [2,4,11,29,30]. We should also consider whether they already had a prenatal subclinical disease.

The poor prognosis might be explained by the delayed diagnosis of breast cancer or by the tendency of tumors to show more aggressive biology [4-6,10,11,15,27]. In our study, breast cancer cases diagnosed during the second and third trimesters were associated with a higher mortality risk (unadjusted HR = 5.9, 90% CI: 1.01 to 35) than those diagnosed during the first trimester, although the difference in diagnosis was to be established. Breast cancer during pregnancy often presents as a painless mass or thickening in the breast. The management of breast cancer during pregnancy should be individualized taking into account maternal health status, TNM stage, and gestational (GA) age at diagnosis [5-7,17,22] as administration of conventional therapies is not possible in all cases [11,22].

Surgery

The second trimester is the safest period for it to be performed, although it may be carried out irrespective of gestational age [5-7,17,22]. In our study, we initially performed a breast-conserving surgery without axillary lymphadenectomy in 12 patients and modified radical mastectomy (MRM) in four cases during pregnancy. After the delivery, the MRM was carried out in six cases, including two cases with local recurrence.

Chemotherapy

Prenatal exposure to chemotherapy may be associated with a higher risk of obstetrical and fetal complications, such as preterm birth, premature rupture of membranes, and intrauterine growth restriction. The transient myelosuppressive effect of chemotherapy affects both the mother and the child. Anthracyclines, taxanes, and alkylating agents can be safely used after the first trimester [5-7,15-17,22], and chemotherapy should be avoided three weeks before delivery. Hormonal therapy and radiotherapy are not recommended at any time during pregnancy [5,6,17,22,24].

In our institution, the upfront chemotherapy was applied in 56% of cases (seven as NC and two as AC) during the second and/or third trimesters with supportive medication (ondansetron and corticosteroids) to reduce side effects. No serious adverse events or deaths were observed in mothers or neonates exposed to chemotherapy [17]. Chemotherapy did not increase neonatal neonatal intensive care unit (NICU) admission rates nor did it reduce birth weight. Only nine patients (56%) received radiotherapy after delivery.

Pregnancy outcomes

For the entire cohort, the median GA at delivery was 37.5 weeks (range: 10-40 weeks); 81.2% of cases ended in a term or preterm pregnancy, and only three cases did it in a miscarriage. In patients who had prenatal exposure to chemotherapy, at least three-quarters of cases (77.7%) ended in a full-term pregnancy and two in preterm delivery, all of this group had a live birth.

There were no antepartum admissions. Delivery after 37 weeks is recommended, avoiding premature delivery whenever possible, and vaginal delivery should be favored over cesarean section [5,6,22]. However, in our study, this was not the case as elective cesarean section was performed in 10 cases and vaginal delivery in three cases. Placental histopathology testing was not performed.

Neonatal outcomes

**Figure 4:**
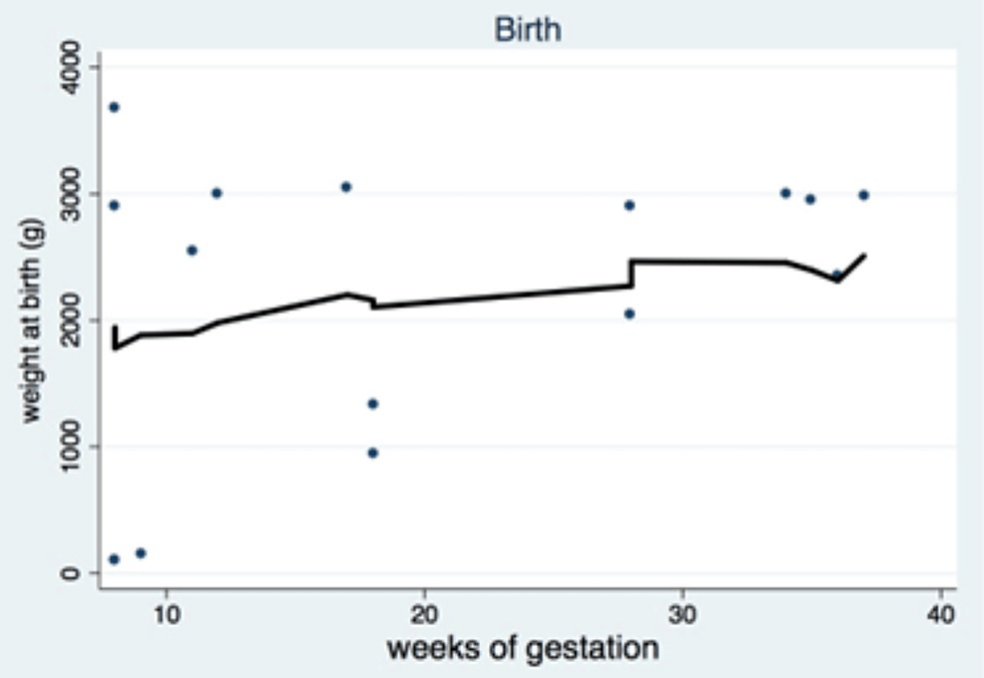
Birth ratio according to the weight at birth and weeks of gestation in pregnant women with breast cancer

Prematurity is the primary cause of many complications in newborns such as respiratory failure, necrotizing enterocolitis, and feeding problems among other complications [5]. In our study, perinatal care was always available. Nine newborns had a birth weight higher than 2,500 g (of this group, four, two, and three cases were diagnosed during the first, second, and third trimesters of pregnancy, respectively). Four newborns had a birth weight below 2,500 g (of this group, three and one cases were diagnosed during the second and third trimesters of pregnancy). An Apgar score > 8 at one minute was recorded in 10 cases, and an Apgar score < 8 was recorded in three cases. Almost all newborns were healthy, except for two cases with respiratory failure, in which one child died due to extreme underweight and prematurity (gestation termination at 31 weeks due to cancer progression), and the other child survived after medical care [17]. When the GA is more than 37 weeks, the child's weight becomes better. In our cohort, this was possible in most cases (Figure 4).

We should recognize obstetric and neonatal risks associated with cancer and the different treatments [22]. Thanks to a multidisciplinary medical team, our institution obtained favorable overall outcomes for both mothers and neonates, with a high percentage of full-term deliveries being obtained, thus reducing the likelihood of NICU admission for preterm newborns. It is a well-known fact that all patients with breast cancer are associated with a high-risk pregnancy at diagnosis. Therefore, pregnant women underwent closer care in order to reduce obstetric and perinatal morbidity. Notwithstanding, PABC itself is a risk factor for preterm birth.

Epidemiological and pathological characteristics as well as the TNM stage are similar to those of other local studies [15]. Our findings showed that women aged ≤35 years with PABC reflected a more aggressive immune-pathological profile, including advanced T/N stage, high grade, ER-/PgR-negative tumors, high-risk NPI and TN tumors, in addition to the fact of delayed diagnosis, all these combined factors act synergistically, which could almost fully explain the poor prognosis. Our data are concordant with other studies that document aggressive tumor characteristics and final prognosis in PABC patients [2-4,6-16,24,27-29].

## Conclusions

This institutional study supports that breast cancer can be relatively safely treated during pregnancy in young women, and closer obstetrical monitoring must be carried out until its conclusion in order to obtain optimal outcomes: healthy mothers with full-term deliveries without complications. Therefore, we should continue for timely detection and better-specialized strategies to maximize health outcomes for the delicate mother-child dyad.
